# Effect of treatment with angiopoietin-2 and vascular endothelial growth factor on the quality of xenografted bovine ovarian tissue in mice

**DOI:** 10.1371/journal.pone.0184546

**Published:** 2017-09-15

**Authors:** Hyun Sun Kong, Jaewang Lee, Hye Won Youm, Seul Ki Kim, Jung Ryeol Lee, Chang Suk Suh, Seok Hyun Kim

**Affiliations:** 1 Department of Obstetrics and Gynecology, Seoul National University Bundang Hospital, Gumi-dong, Bundang-gu, Seongnam, Korea; 2 Department of Obstetrics and Gynecology, Seoul National University College of Medicine, Seoul, Korea; 3 Department of Obstetrics and Gynecology, Seoul National University Hospital, Seoul, Korea; Faculty of Animal Sciences and Food Engineering, University of São Paulo, BRAZIL

## Abstract

Cryopreservation and transplantation of ovarian tissue (OT) represents a method for fertility preservation. However, as the transplantation is performed without vessel anastomosis, unavoidable ischemic damage occurs. To reduce this ischemic damage and improve outcomes after transplantation, we used two kind of angiogenic factors, angiopoietin-2 (ang-2) and vascular endothelial growth factor (VEGF). Fresh or vitrified-warmed bovine OTs were prepared for xenotransplantation (XT). Fresh OTs were immediately xenografted into nude mice (XT-Fresh). Vitrified-warmed OTs were xenografted into four subgroups of mice, which were injected intraperitoneally before XT with saline (XT-Vitri), Ang-2 (XT-Ang-2), VEGF (XT-VEGF), and a combination of Ang-2 and VEGF (XT-Combined). Seven or 28 days post-grafting, grafted OTs and blood samples were collected for evaluation. Follicle normality was higher in the angiogenic factor-treated groups than in the XT-Vitri group. The XT-VEGF and the XT-Combined showed higher (P<0.05) follicular density than the XT-Vitri group. The highest apoptotic follicle ratio was observed in the XT-Vitri group on day 7; this was decreased (P<0.05) in the XT-Combined group. Microvessel densities were higher in the angiogenic factor-treated groups than in the XT-Vitri group. The largest fibrotic area was showed in the XT-Vitri group on day 28, and it was decreased (P<0.05) in the XT-combined group. Based on these results, administration of Ang-2 and VEGF to recipients prior to XT appeared to alleviate ischemic damage by enhancing angiogenesis, which resulted in the maintenance of follicle integrity and density, and reduced follicle apoptosis and OT fibrosis.

## Introduction

Advances in cancer therapy have improved survival outcomes in patients with cancer. However, reduced fertility, with premature ovarian failure, is often observed in young women following treatment for cancer [1, 2]. Several options, such as oocyte/embryo or ovarian tissue (OT) cryopreservation [3], are available for fertility preservation in such patients. OT cryopreservation represents the best option for fertility preservation in pre-pubertal girls and patients who cannot delay chemotherapy. To date, over 86 babies have been born via this technique [4]; however, the procedure is still considered to be at an innovative treatment.

The quality of OT grafts may be affected by several factors; ischemic injury following transplantation is a major contributor to ovarian follicle depletion and poor stromal cell quality [5, 6]. According to several reports, neovascularization occurs within 48 hours after grafting in rats [7], 1 week in sheep [8, 9], and 5 days in humans [10]. Before the completion of neovascularization, the OT grafts are subject to ischemic and hypoxic environments. In order to overcome ischemic injury and achieve successful OT function, it is necessary to shorten the ischemic period and promote neovascularization. Therefore, increased efforts have been made to promote neovascularization during the early ischemic period. Numerous studies have attempted to determine the most suitable grafting site [11, 12], optimal ovarian cortex size [3], and to identify effective treatment substances for enhancement of neovascularization to achieve OT graft survival and restore ovarian function [13–16]. However, optimization of this technique is still necessary to improve outcomes following transplantation.

Vascular endothelial growth factor (VEGF), a potent angiogenic factor, has been utilized in several studies to enhance neovascularization following auto- or xenotransplantation (XT) of OTs in mice, rabbits, and humans [13, 14, 17]. Angiopoietin (Ang) family proteins are additional angiogenic growth factors, including Ang-1 and Ang-2 [18]. Ang-1 has been shown to play a role in vessel stabilization during the normoxia phase, and Ang-2 in postnatal angiogenesis during the hypoxia phase [19–21]. In particular, Ang-2 has been reported to participate in the wound healing process, with high levels of Ang-2 and VEGF occurring at hypoxic and ischemic sites [22, 23]. Our previous study demonstrated that the treatment of mice with Ang-2 after transplantation of cryopreserved OTs had beneficial effects in terms of facilitating neovascularization of OT grafts [24] Furthermore, Asahara et al. reported that the co-administration of Ang-2 and VEGF induced greater levels of vessel formation in the eye compared with that after administration of Ang-2 or VEGF alone [25]. However, to our knowledge, there has been no previous study of the effect of combined treatment with Ang-2 and VEGF on neovascularization following OT transplantation.

The present study was performed to evaluate the effects of angiogenic factors Ang-2 and VEGF, administered alone and in combination, on the improvement of XT of bovine OT graft survival and quality via enhancement of neovascularization and reduction of ischemic injury.

## Materials and methods

### Study design

**Fig 1:**
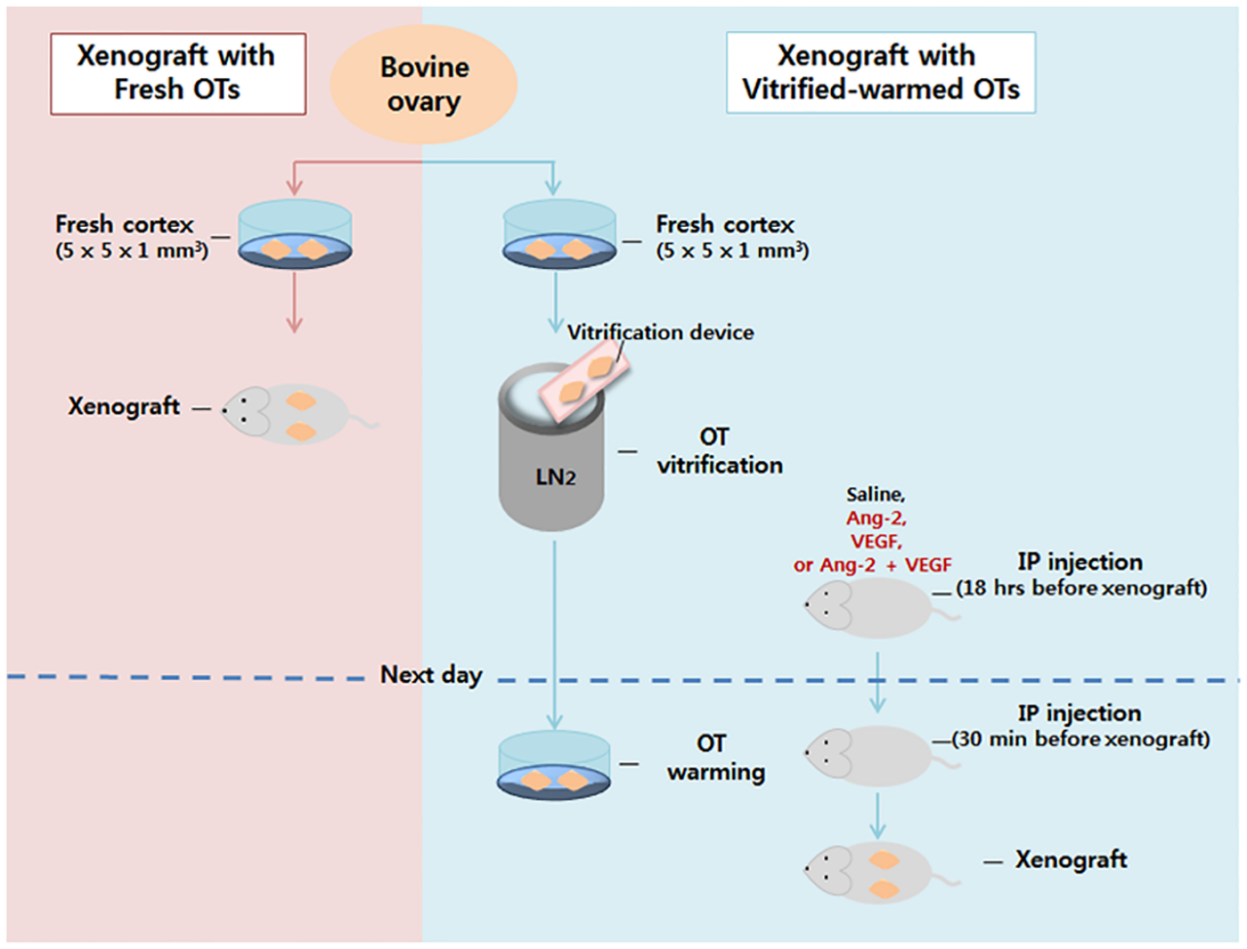
The experimental scheme. Xenografting of fresh and vitrified-warmed ovarian tissues is described on the left and right side of the figure, respectively. OT: ovarian tissue, IP: intraperitoneal injection.

The experimental scheme is shown in Fig 1. Briefly, bovine OT preparation and cryopreservation, administration of angiogenic factors or normal saline to mice, and the xenografting procedure and the timescale for the experiments are shown. After the procedure, the mice were sacrificed for graft retrieval and sample analysis.

### Preparation of bovine ovarian tissue

Ovaries were obtained from 40- to 70-month-old bovines (n = 16). The ovaries immersed in Leibovitz’s L-15 (L-15) medium (WelGene, Daegu, Korea) at 37°C were transported from a local slaughterhouse to our laboratory within 2 hours. The ovaries were swabbed with 70% alcohol to eliminate blood and other contaminants, and then washed once more with L-15 medium. Next, each ovary was cut in half and the medullar region was removed using curved scissors and forceps. Then, the OT cortex was sliced into portions of 5 × 5 × 1 mm^3^ in size; some of the fresh OT samples obtained were used for fresh OT (without vitrification) transplantation and the rest were vitrified for subsequent experiments.

### Vitrification and warming

As previously described by Youm et al., the OTs were vitrified using a two-step vitrification method with slight modification [12]. Briefly, the OTs were submerged in 7.5% ethylene glycol (EG; Sigma-Aldrich, Missouri, US) and 7.5% dimethyl sulfoxide (DMSO; Sigma-Aldrich, Missouri, US) in L-15 medium with 20% fetal bovine serum (FBS; Gibco, Paisley, UK) for 15 minutes at room temperature (RT). For the second step, the OTs were transferred into a solution containing 20% EG, 20% DMSO, and 0.5 M of sucrose (Sigma-Aldrich, Missouri, US) in L-15 medium, with 20% FBS, for 10 minutes at RT. Immediately after the second step, the samples were placed on a metallic cryopreservation device (Cryotissue; Kitazato BioPharma, Shizuoka, Japan) and directly plunged into liquid nitrogen (LN_2_), and stored in an LN_2_ tank for one day.

For warming, the OTs were processed in serially diluted medium containing 1 M, 0.5 M, 0.25 M, and 0 M of sucrose in L-15 medium with 20% FBS. The first step was performed for 1 minute at 37°C, and the other steps for 5 minutes each at RT.

### Xenotransplantation into nude mice

Nine-week-old BALB/c nude mice (Orient Co., Seoul, South Korea), housed under a 12-hour light/dark cycle at 22°C and fed ad libitum, were used in this study. All experimental procedures were approved by the Institutional Animal Care and Use Committee of Seoul National University Bundang Hospital (IACUC approval number: BA1402-147/008-01). The nude mice were anesthetized by intraperitoneal injection of a mixture of 30 mg/kg zolazepam + tiletamine and 10 mg/kg xylazine. For mouse euthanasia, cervical dislocation was performed by experienced specialist.

For XT, two slices of OT were transplanted per one nude mouse. The nude mice (n = 55) were randomly divided into 5 XT groups, out of which one group was xenotransplanted with non-vitrified fresh OT (XT-Fresh; n = 11). In the other four groups, nude mice were xenotransplanted with vitrified-warmed OTs. Eighteen hours and 30 minutes before xenotransplantation with vitrified-warmed OTs, each group was intraperitoneally injected with one of the following: saline (XT-Vitri; n = 11), 500 ng of Ang-2 (R&D systems, Minnesota, USA) (XT-Ang-2; n = 11), 200 ng of VEGF (R&D systems, Minnesota, USA) (XT-VEGF; n = 12), and a combination of 500 ng of Ang-2 and 200 ng of VEGF (XT-Combined; n = 10).

For removal of mouse ovaries and XT of bovine OT, the nude mice were anesthetized and bilateral ovariectomy was performed in each mouse by making a small incision in the middle dorsal part along the spinal line [26]. Then, the bovine OT samples (5 × 5 × 1 mm^3^) were transplanted into the dorsal subcutaneous sites, and the OTs were sutured using 5–0 nylon. The skin wounds were clipped using a 9-mm auto clip (Jeungdo Bio & Plant, Seoul, Korea), and gentamicin (0.75 mg/mouse) was injected intraperitoneally.

### Graft retrieval

The nude mice were sacrificed by cervical dislocation and the bovine OT grafts were retrieved 7 or 28 days after grafting. The retrieved OT grafts were washed in normal saline several times after elimination of attached tissue. Then, the OT grafts were immediately fixed in Bouin’s solution (Sigma-Aldrich, Missouri, US) for one day, followed by embedding in paraffin block for histological analysis.

### Histological analysis

The bovine OT grafts embedded in paraffin blocks were serially sectioned at 5-μm thickness. Every 10th section was stained with hematoxylin (DAKO, Seoul, Korea) and eosin (Merck, Darmstadt, Germany) (H&E), and three sections per graft were chosen for follicle grading. The rest were used for immunostaining.

The H&E-stained slides were blindly read twice by a single experienced inspector for measurement of follicular stage, normality and density using a light microscope (Nikon, Tokyo, Japan). The developmental stages of follicles were classified according to the following categories [27]: (1) primordial follicles: single layer of flattened pre-granulosa cells; (2) primary follicles: single layer of cuboidal granulosa cells; (3) secondary follicles: two or more layers of cuboidal granulosa cells, with the antrum absent; (4) antral follicles: multiple layers of cuboidal granulosa cells, with the antrum present.

For follicle normality measurement, follicles of the total H&E-stained area were evaluated. Since the 1-mm-thick ovarian cortical tissue mainly contains primordial follicles, primordial or primary follicles are mainly observed after the xenotransplantation [28, 29]. Thus, the follicles were categorized as primordial and growing follicles instead of primordial, primary, secondary and antral follicles during the morphological analysis. Only follicles with a clearly visible oocyte encircled by a granulosa cell layer were counted, and the follicles were classified as morphologically normal or degenerated. Follicles were considered degenerated if they had pyknotic bodies within granulosa cells, condensed oocyte nuclei, shrunken oocytes, oocyte cytoplasm vacuolization, or low cellular density [30]. For measurement of follicular density, follicles were examined in randomly selected three high-power fields (HPF; magnification: 400 ×; 72900 μm^2^) per section which was chosen for follicle grading.

### Follicular apoptosis

Apoptosis of the ovarian follicles was detected using an In Situ Cell Death Detection Kit (Roche, Basel, Switzerland) as previously described by us [31]. OTs were mounted using VECTASHIELD Mounting Medium with 4',6-diamidino-2-phenylindole (Vector Laboratories, California, USA) and visualized under an inverted Zeiss AX10 fluorescence microscope (Carl Zeiss, Oberkochen, Germany); cells with fragmented DNA displayed green fluorescence and normal cells, which were counterstained, displayed blue fluorescence. Follicles with over 30% apoptosis-positive cells (emitting a green signal) were categorized apoptotic follicles [12, 31–33].

### Immunohistochemistry

CD31 immunostaining of the grafts was performed to detect microvessels. First, the paraffinized OT slides were deparaffinized and rehydrated in xylene and ethanol, respectively. For target antigen retrieval, the rehydrated slides were microwaved (700 watts) with pH 9.0 Tris/EDTA buffer (DAKO, Seoul, Korea) for 20 minutes. After cooling, the slides were treated with peroxidase-blocking solution (DAKO, Seoul, Korea) for 10 minutes, followed by incubation with CD31 antibody (1: 400, Bioss, Massachusetts, USA) for 1 hour at RT. After treatment with the primary antibody (CD31), the slides were washed and treated with EnVision/HRP solution (DAKO, Seoul, Korea) for 30 minutes, and subsequently with substrate-chromogen solution (DAKO, Seoul, Korea) for 10 minutes. All slides were counterstained with hematoxylin (DAKO, Seoul, Korea), and the slides were investigated in blinded fashion. CD31-positive microvessels were randomly counted in 3 HFPs (400 ×; 72900 μm^2^) per graft under a light microscope (Nikon, Tokyo, Japan).

### Evaluation of graft fibrosis

In order to evaluate the fibrotic surface area, Masson’s trichrome staining was performed using the Roche Trichrome III Blue Staining Kit (Roche, Basel, Switzerland). The fibrotic surface, nuclei, and cytoplasm were stained blue, black, and red, respectively. The slides were visualized by light microscopy at 100× magnification, and fibrosis relative surface area were analyzed using i-Solution image analysis software (IMT i-Solution Inc., Daejeon, Korea).

### Hormonal assay

Blood samples were obtained from recipient mice after sacrifice, and sera were separated from the blood samples by centrifugation for 2 minutes at 13,000 rpm and 4°C. The sera were stored at −80°C for measurement of bovine estradiol levels using a bovine estradiol Enzyme-Linked Immunosorbent Assay kit (Cusabio, Wuhan, China).

### Statistical analyses

The chi-square test was used to analyze the effect of each treatment on follicular normality and apoptosis ratio. The follicle and microvessel density, graft fibrosis, and hormonal assay data were analyzed by one-way analysis of variance (ANOVA). Statistical software package SPSS 18.0 (SPSS Inc., Chicago, USA) and GraphPad Prism version 6.0 were used (Graph-Pad, San Diego, CA, USA). A probability of P < 0.05 indicated that a difference was significant.

## Results

### Histological evaluation

**Fig 2:**
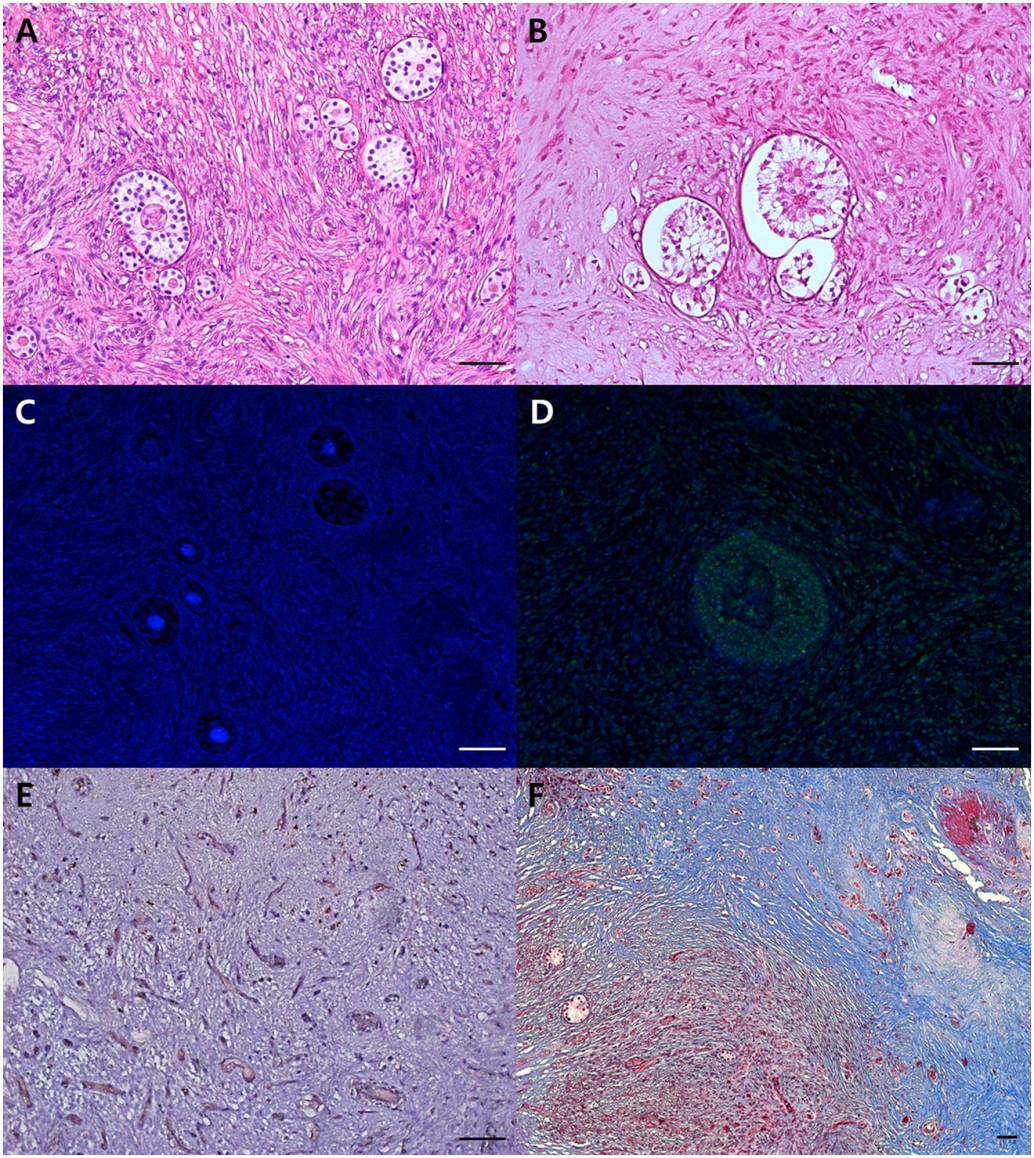
Representative images of ovarian grafts. Each panel shows (A) H&E-stained graft with morphologically normal follicles, (B) H&E-stained graft with morphologically abnormal follicles (follicle shrinkage and rupture, oocyte rupture), (C) TUNEL-stained graft with non-apoptotic follicle (blue), (D) TUNEL-stained graft with apoptotic follicle (green), (E) CD31-immunostained microvessels (brown), and (F) Masson’s trichrome-stained graft (red: cytoplasm, blue: fibrotic area, black: nuclei). Scale bar: 100 μm.

As shown in Fig 2A and 2B, morphologically normal and abnormal follicles were evaluated. A total of 6,658 follicles (day 7: 3,896, day 28: 2,762) were analyzed for morphology; the results are shown in Tables 1 and 2. Regardless of follicular developmental status, the proportion of morphologically normal follicles was lower (P < 0.05) in the XT-Vitri group than in the XT-Fresh group on day 7 (Table 1), but significantly higher in the angiogenic factor-treated groups than in the XT-Vitri group.

**Table 1:** Follicular normality, density, and apoptotic ratio in accordance with different xenotransplantation groups after 7 days of grafting.

Groups	Grafts No.	Follicular normality			Follicular density (/HPF)			Apoptotic follicle
		Primordial	Growing	Total	Primordial	Growing	Total	
XT-Fresh	10	76.0% (430/566)^a^	71.1% (384/540)^a^	73.6% (814/1106)^a^	2.0±0.2^a^	1.9±0.2^a^	3.9±0.4^a^	12.0% (32/266)^ab^
XT-Vitri	10	55.1% (147/267)^b^	55.2% (133/241)^b^	55.1% (280/508)^b^	0.9±0.1^b^	1.0±0.1^bc^	1.9±0.2^b^	18.7% (20/107)^a^
XT-Ang-2	10	64.7% (209/323)^c^	64.4% (199/309)^c^	64.6% (408/632)^c^	1.2±0.1^bc^	1.4±0.2^ac^	2.6±0.2^bc^	12.9% (20/155)^ab^
XT-VEGF	12	63.2% (278/440)^c^	68.0% (272/400)^ac^	65.5% (550/840)^c^	1.1±0.1^bc^	1.8±0.1^a^	2.9±0.2^c^	13.4% (37/277)^ab^
XT-Combined	10	67.1% (278/414)^c^	64.6% (256/396)^c^	65.9% (534/810)^c^	1.5±0.2^ac^	1.9±0.2^a^	3.4±0.3^ac^	9.4% (19/203)^b^

Different superscript means a statistical significance within the same column.

HPF: high power field (72900 μm^2^)

**Table 2:** Follicular normality, density, and apoptotic ratio in accordance with different xenotransplantation groups after 28 days of grafting.

Groups	Grafts No.	Follicular normality			Follicular density (/HPF)			Apoptotic follicle
		Primordial	Growing	Total	Primordial	Growing	Total	
XT-Fresh	12	73.9% (139/188)^a^	82.6% (347/420)^a^	79.9% (486/608)^a^	1.0±0.1^ab^	2.4±0.3	3.3±0.3^ab^	10.1% (17/169)
XT-Vitri	12	48.6% (51/105)^b^	77.7% (251/323)^ac^	70.6% (302/428)^b^	0.6±0.1^a^	1.8±0.2	2.4±0.2^a^	5.8% (8/139)
XT-Ang-2	12	65.6% (101/154)^a^	73.3% (275/375)^bc^	71.1% (376/529)^b^	0.8±0.1^a^	2.3±0.3	3.1±0.3^ab^	4.4% (7/158)
XT-VEGF	12	70.5% (105/149)^a^	79.8% (344/431)^ac^	77.4% (449/580)^a^	0.9±0.1^a^	2.4±0.2	3.3±0.3^ab^	8.9% (27/302)
XT-Combined	10	69.7% (129/185)^a^	83.6% (361/432)^a^	79.4% (490/617)^a^	1.5±0.2^b^	2.5±0.3	4.0±0.4^b^	7.0% (9/128)

Different superscript means a statistical significance within the same column.

HPF: high power field (72900 μm^2^)

On day 28, the proportion of morphologically normal primordial follicles in XT-Vitri group was lower (P < 0.05) than in the XT-Fresh group. However, this proportion was higher in the angiogenic factor-treated groups, and similar to that of the XT-fresh group (Table 2). With regard to growing follicles, normality of the XT-Vitri group was similar to that of the XT-Fresh group and the angiogenic factor treated-groups. In addition, the follicular normality of the XT-VEGF and XT-Combined group was similar to that of the XT-Fresh group had.

### Ovarian follicle density

A decrease (P < 0.05) in the density of primordial and growing follicles, and in the total follicle density was observed in the XT-Vitri group relative to the XT-Fresh group on day 7 (Table 1). Compared with the XT-Vitri group, the density of these follicles was higher (P < 0.05) in the in XT-Combined group. In the XT-VEGF group, an increase (P < 0.05) in the density of growing follicles, as well as in the total follicle density, was observed when compared with the XT-Vitri group.

On day 28, the lowest primordial follicle density was observed in the XT-Vitri group when compared with the XT-Combined group. The XT-Combined group showed similar primordial follicle density to the XT-Fresh group (Table 2). The density of growing follicles was similar (P > 0.05) among the treatments. With regard to total follicle density, the XT-Vitri group showed the lowest count, which was significantly different from that of the XT-Combined group.

### Follicle apoptosis

As shown in Fig 2C and 2D, apoptotic and non-apoptotic follicles were counted to measure the proportion of apoptotic follicles. Compare to the XT-Vitri group, the XT-Combined group showed significantly lower (P < 0.05) apoptotic follicle ratio on day 7 (Table 1). There was no difference in the proportions of apoptotic follicles between the groups on day 28 (Table 2).

### Microvessel density

**Fig 3:**
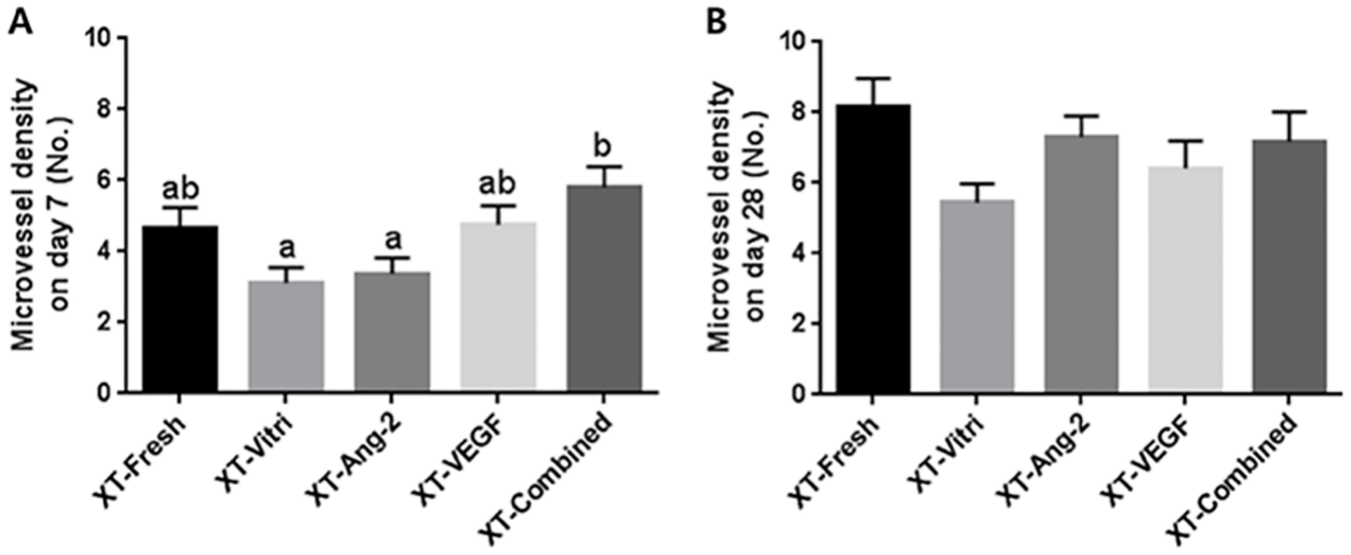
Densities of CD31 (+)-stained microvessels. Each graph indicates microvessel densities on day 7 (A) and day 28 (B) after xenografting of bovine ovarian tissue. Noncommon superscript letters differ (P < 0.05).

CD31-positive microvessels were counted in order to evaluate microvessel density, as shown in Fig 2E. On day 7, the lowest mean number of microvessels was observed in the XT-Vitri group when compared with the XT-Combined group (Fig 3A). On day 28, the microvessel densities were similar (P > 0.05) among the treatments (Fig 3B).

### Evaluation of graft fibrosis

**Fig 4:**
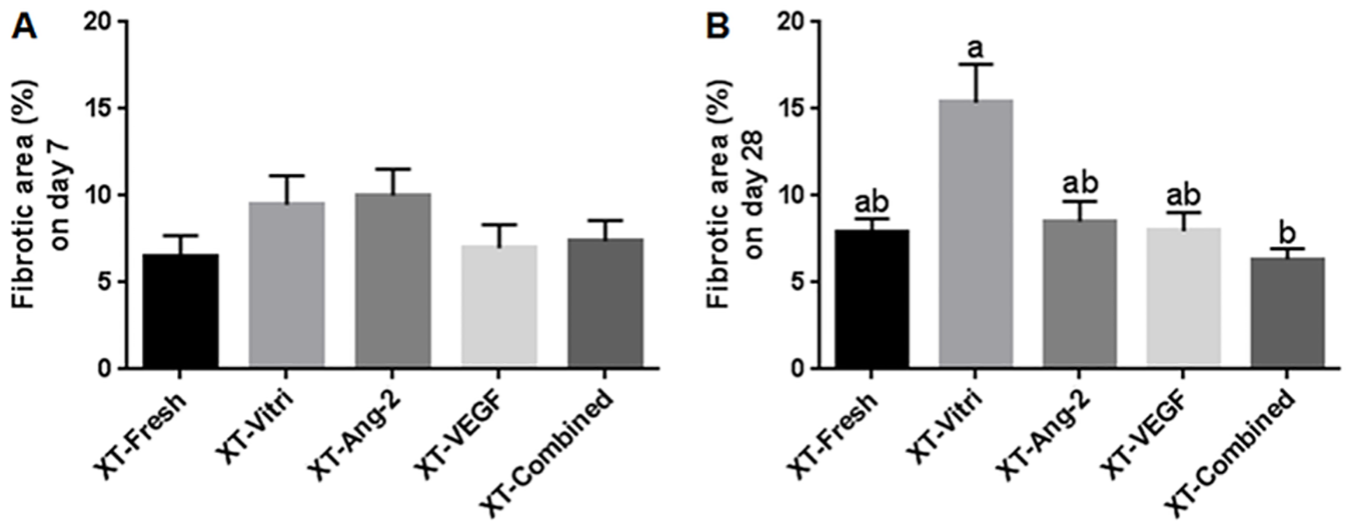
Fibrosis relative surface area of the grafts. Each graph represents the fibrotic surface ratio of the graft on day 7 (A) and on day 28 (B) after xenografting of bovine ovarian tissue. Noncommon superscript letters differ (P < 0.05).

A representative picture of a fibrotic graft is shown in Fig 2F. The fibrotic area (stained blue) was evaluated, and there were no significant differences between the groups on day 7 (Fig 4A). On day 28, graft fibrosis area was smaller (P < 0.05) in the XT-Combined group (6.3 ± 0.6) compared with the XT-Vitri group (7.9 ± 0.8; Fig 4B).

### Serum estradiol levels

With regard to the serum estradiol levels, no significant differences between the groups were observed on day 7 and 28. The hormone levels for each group on day 28 (XT-Fresh: 102.3 ± 13.7; XT-Vitri: 95.3 ± 6.0; XT-Ang-2: 95.2 ± 7.7; XT-VEGF: 95.4 ± 11.2; XT-Combined: 115.5 ± 8.5) were similar to those on day 7 (XT-Fresh: 107.8 ± 6.9; XT-Vitri: 105.7 ± 20.8; XT-Ang-2: 112.6 ± 15.6; XT-VEGF: 101.5 ± 11.9; XT-Combined: 97.6 ± 8.2).

## Discussion

In this study, angiogenic factors Ang-2 and/or VEGF were injected into recipient mice to enhance angiogenesis and reduce ischemic injury following the XT of avascular bovine OT. Higher microvessel densities with improved follicle morphology and densities, as well as lower apoptotic follicle ratios, were observed in the angiogenic factor-treated groups compared to the saline-treated XT-Vitri group 7 days after grafting. Furthermore, the angiogenic factor-treated groups showed improved follicle morphology and density and lower graft fibrosis than the XT-Vitri group on day 28.

The dose of Ang-2 was based on our previous study of the effect of Ang-2 on autotransplanted mouse ovary [24]. Treatment with mouse VEGF_164_ isoform elicited an improvement in neovascularization, according to previous studies [11]; therefore, VEGF_164_ was utilized in this study. The effective dose of VEGF for injection in the mouse model was based on a previous study by Hiratsuka et al. [23]. According to Ang-2 half-life, Ang-2 was injected to recipient 18 hours and 30 minutes (injected twice) before vitrified mouse OT autotransplantation to facilitate neovascularization [24]. The exact half-life of VEGF_164_ used in this study is not known, but VEGF is known to have short half-life [34]. Even though VEGF has short half-life, it shows its angiogenic effect in previous studies [35–37]. Since the half-life of Ang-2 seems longer than that of VEGF, injection timing before XT was determined based on the half-life of Ang-2 being 18 hours, and the angiogenic factors were injected once again before the XT procedures.

Regardless of the day of graft retrieval, follicle normality in the XT-Vitri group was lower (P < 0.05) than that in the XT-Fresh group. Cryopreservation of OT results in unavoidable cryodamage to the samples, which is aggravated by the avascular grafting procedure, resulting in an even greater deterioration of OT quality. Similar phenomena have been reported by previous OT xenografting studies [38, 39]. In contrast with the poor outcomes of follicle normality in the XT-Vitri group, higher follicle normality was observed in the angiogenic factor-treated groups on day 7. Except for growing follicle normality of XT-Ang-2 group, higher follicle normality was observed in the angiogenic factor-treated groups than that of XT-Vitri group on day 28. We hypothesize that the increase in microvessel density, modulated by exogenous angiogenic factors, exerts beneficial effects on follicle morphology of the cryopreserved OT grafts. A gradual increase in microvessel density was observed in the XT-Ang-2, XT-VEGF, and XT-Combined groups in the early post-transplantation period (day 7). Therefore, we assumed that relatively low numbers of microvessels in the XT-Ang-2 group, on day 7, resulted in the low growing follicle normality observed on day 28.

A significant reduction in the follicle density of cryopreserved grafts has been reported in previous studies [29, 38], which is consistent with the present results for the XT-Vitri group relative to the XT-Fresh group. However, among the groups xenografted with vitrified-warmed OT, the primordial follicle density of the XT-Combined group was higher (P < 0.05) on day 7 than that of the XT-Vitri group. In addition, the density of growing follicles was higher (P < 0.05) in the XT-VEGF and XT-Combined groups than in the XT-Vitri group. The increasing trend for follicle density in the angiogenic factor-treated groups was also observed for microvessel density. Consistent with our findings, Wu et al. showed that the density of primordial and primary follicles was higher in well-vascularized grafts treated with *Salviae miltiorrhizae* than in non-treated cryopreserved OT grafts [40]. Therefore, we propose that treatment with angiogenic factors results in the acceleration of angiogenesis and prevents follicle loss; our findings indicate that Ang-2 /VEGF co-treatment achieved the highest improvement of follicle density among the angiogenic factor-treated groups. On day 28, the primordial follicle density of the XT-Combined group was higher (P < 0.05) than that of the XT-Vitri group. As the graft stabilized on day 28, the significance of the difference between the XT-Vitri and angiogenic factor-treated groups, in terms of density of growing follicles, disappeared; however, the total follicle density trend on day 28 was similar to that observed on day 7. Thus, the finding confirms that the improved microvessel density elicits an increase in graft follicle density, and that this beneficial effect may be observed up to 28 days following transplantation.

Compared with the XT-Vitri group on day 7, lower apoptotic follicle ratios were observed in the angiogenic factor-treated groups; in particular, these were significantly reduced in the XT-Combined group. In a previous study by Wang et al., xenografted human OT co-treated with VEGF and bFGF showed significantly reduced graft apoptosis, with increased vessel density, relative to the control (fresh OT graft) [25]; these findings were similar to those of the present study. The authors attributed the reduction in graft apoptosis to the high level of neovascularization post-grafting. Our study additionally demonstrated that increased angiogenesis after grafting appeared to alleviate follicle apoptosis 7 days following grafting; however, the significance disappeared 28 days after grafting. Previous studies demonstrated that graft apoptosis levels are relatively high during the early post-transplantation period; however, these levels decrease as the graft stabilizes and vascularization occurs [41–43]. Based on previous studies [41–43], we suggest that the grafted OTs in all of the present groups were stabilized by day 28; therefore, no significant difference in follicle apoptosis was observed between the groups at this stage.

In the present study, OTs grafted following cryopreservation showed lower (P < 0.05) microvessel density than fresh grafts. The reduced numbers of microvessels in the cryopreserved grafts may be attributed to unavoidable cryodamage. The cell count and proliferation rates of human saphenous vein endothelial cells, following cryopreservation and in vitro culture, were found to be lower than those of fresh endothelial cells [44]. Accordingly, the reduced proliferation rate of endothelial cells following cryopreservation may explain the lower numbers of microvessels after XT of grafts. However, in this study, treatment with angiogenic factors elicited an elevation in microvessel density of the cryopreserved OT after grafting, compared to that of the XT-Vitri group. These results suggest that treatment with angiogenic factors, particularly co-treatment with Ang-2 and VEGF, compensates for the harmful effects of cryopreservation on graft quality and promotes the revascularization of graft tissue more effectively.

Ang-2 and VEGF play different roles in angiogenesis: VEGF promotes angiogenesis by activating endothelial cell proliferation and migration [20]; however, Ang-2, which does not play a mitogenic role in endothelial cells, promotes vessel destabilization to initiate neovascularization and facilitate the activity of other endothelial-acting cytokines such as VEGF [21, 22]. Accordingly, the Ang-2 treated group showed slightly similar microvessel densities to the XT-Vitri group on day 7, as Ang-2 did not play a role in endothelial cell proliferation. Asahara et al. reported that an increase in microvessel density was not observed following the injection of Ang-2 into the mouse eye (for corneal micropocket assay), which is consistent with our results [45]. However, higher microvessel densities were observed in the XT-VEGF and XT-Combined group than in the XT-Vitri and XT-Ang-2 groups on day 7. In particular, the highest microvessel density was observed in the XT-Combined group, and the difference was significant. This result agrees with the study of Asahara et al. in that co-treatment with Ang-2 and VEGF elicits a higher degree of vascularization in the mouse eye than treatment with Ang-2 or VEGF only. Therefore, in the present study, it was considered that the modulatory effect of exogenous Ang-2 on VEGF and other cytokines resulted in the highest microvessel density observed in the XT-Combined group on day 7. As the grafts were well-stabilized on day 28, no significant difference was observed in the microvessel densities of the groups at this stage.

Several studies have reported the occurrence of extensive fibrosis, resulting from ischemic damage, following XT [46, 47]. In the present study, significant differences between the fibrotic area were not observed between the the groups on day 7; however, on day 28, the XT-Vitri group showed a higher (P < 0.05) fibrotic ratio than the XT-Combined group. We speculate that decreased (P < 0.05) fibrotic ratio in the XT-Combined group on day 28 resulted from the enhanced angiogenesis elicited by co-treatment with Ang-2 and VEGF. The observed reduction in fibrotic area in the angiogenic factor-treated groups in the present study, especially in the XT-Combined group, is consistent with the report of Wang et al. which described a significant decrease in fibrosis in human OT grafts after injection of the recipients with angiogenic factors (VEGF or co-treatment with VEGF and bFGF) [25].

No significant differences in the levels of bovine serum estradiol were observed in this study. Estradiol level is known to correlate to the ovarian follicular pool and size. In the present study, we used 1-mm-thick ovarian cortex samples, which mainly contained primordial follicles. When examined 7 days or 28 days post-grafting, the grafts were found to consist of small growing follicles, which mainly represented primary follicles. This finding suggests that the grafting periods of 7 and 28 days may not be sufficient to fully observe significant hormonal change.

In this study, three sections per graft and three high-power fields per section were evaluated for follicle normality and density, respectively. Ovarian follicle distribution is known to be heterogeneous [48, 49]. Three sections per graft and three HPFs per section might be not enough to overcome the bias from ovarian follicle heterogeneity and that could be a limitation of our study. However, to minimize the heterogeneity bias, we used one ovary for one round of experiment. The OTs excised from a single ovary were randomly divided into all 5 groups and xenotransplanted to the recipient mice. Additionally, by choosing three sections per one graft, a total of 30 to 36 sections and 90 to 108 HPFs were evaluated for one group. As a result, we have analyzed a total of 3896 follicles for the day 7 groups and 2762 follicles for the day 28 groups. Therefore, we thought that potential bias has been minimized in this study.

To our knowledge, this is the first study that documents the combined effect of Ang-2 and VEGF on xenografted OTs. Our results show that the administration of Ang-2 and VEGF to recipients prior to XT appears to alleviate ischemic damage by enhancing angiogenesis, which results in the maintenance of follicle normality and density, and reduces levels of apoptosis and fibrosis in the grafts. However, further studies involving localized treatment with angiogenic factors are needed in order to develop our findings for application in humans.

## Supporting information

S1 File Supplementary raw data of this experiment. (XLSX)
